# In this month’s Bulletin

**DOI:** 10.2471/BLT.23.001223

**Published:** 2023-12-01

In the editorial section, Manjulaa Narasimhan et al. (750) call for papers for a theme issue on sexual health and well-being across the life course. Alan D Lopez et al. (751) argue that health sector leadership is needed to improve civil registration and vital statistics systems.

Gary Humphreys (754–755) reports on the potential public health implications of a proposed global plastics treaty and interviews Rabih El Chammay (756–757) about the importance of mental health in humanitarian emergencies.

## Afghanistan, Albania, Andorra, Bahamas, Bahrain, Cameroon, Cuba, Ethiopia, Fiji, Georgia, Honduras, Iraq, Jordan, Kazakhstan, Kenya, Kyrgyzstan, Lao People's Democratic Republic, Liechtenstein, Maldives, Montenegro, Morocco, Paraguay, Qatar, Tajikistan, Turkmenistan, Trinidad and Tobago, Uganda, Ukraine, Uzbekistan, Viet Nam

### Tracking air quality indicators

Kerolyn Shairsingh et al. (800–807) introduce new countries in WHO’s database.

## Yemen

### Polio outbreak response

Ibrahem Abduallah Beshr et al. (808–812) track vaccination campaigns.

## Global

### Evaluating population data sources

Lene Mikkelsen et al (758–767) compare the performance of national civil registration and vital statistics systems.

Tim Adair et al. present a global analysis of birth statistics (768–776) and assess the policy utility of routine mortality statistics (777–785). 

### Investing in eye health

Brad Wong et al. (786–799) review the evidence of economic benefits.

